# Challenges for radiologists dealing with clinical decision support systems (CDSS) from a legal point of view

**DOI:** 10.1007/s00330-023-10206-0

**Published:** 2023-09-22

**Authors:** Britta Rosenberg

**Affiliations:** Institute of Diagnostic Radiology and Neuroradiology, University Medicine Greifswald, Greifswald, Germany

During the diagnostic process, radiological examinations are typically requested by clinical physicians. Radiologists check the imaging indication and then approve the examination or refuse it. In the case of examinations that use ionizing radiation (X-rays, computed tomography), based on European law, the so-called justifying indication has to be provided by authorized physicians, in most cases radiologists [1]. The advantages of each examination for the patient (possibility of a better diagnosis) are individually weighed against disadvantages (radiation exposure, possible induction of carcinomas). Regarding ultrasound, an examination without ionizing radiation, the disadvantages of an unnecessary examination are not of a medical nature (radiation exposure or damage to the patient), but rather economic ones (misallocation of resources). Occasional conflicts between the radiologist and the referring clinician are inevitable. This is a hot topic particularly in emergency departments, and during night shifts when time is essential, and the level of experience may not be the highest among both the ordering physicians and on-call radiologists as well. Guidelines, i.e., rule-based lists that assign justified examinations to certain symptoms, signs and suspected diagnoses, were created to help alleviate these conditions.

The European Society of Radiology (ESR) has developed a Clinical Decision Support System (CDSS) as referral guidelines for Europe (ESR-iGuide) embedded into an online web portal [2]. ESR-iGuide recommends the most appropriate imaging tests based on patient data, together with their level of appropriateness, estimated cost, and expected radiation exposure. The tool was developed in 2014 and is based on the guidelines of American College of Radiology (ACR) adapted for European guidelines [3]. In the USA, CDSS are implemented into clinical care through reimbursement guidelines [4]. This of course serves as a strong incentive to comply with the recommendations of CDSS.

The article “Inappropriate CT examinations: how much, who and where? Insights from a clinical decision support system (CDSS) analysis”, published in *European Radiology*, is based on a study of the ESR-iGuide’s benefits in practical application [5].

Authors Rosen, Singer, Vaknin et al. compared examinations which had been indicated and carried out in everyday clinical practice with the examinations that the CDSS of the ESR would have recommended in these situations. Differences were correlated with typical correlations in hospital work (less experienced/very experienced referring physicians; day shift/emergency service; surgical/non-surgical referring physicians). The study design of the trial commented on here did not aim at nor allow any conclusions about missed diagnoses due to refusal of examinations: the study examined a plus of examinations. One interesting result among several was that very experienced referring physicians were particularly prone to non-standard requests. It is easy to imagine that their remembering of prior missed diagnoses could lead to such demands. From the point of view of a lawyer who has been working in the hospital for years, the following considerations on the use of a CDSS arise:

Although the usage of CDSS especially in emergency department and during night shifts is very useful for acute management, like the study shows, it has an inherent low risk for missed diagnoses or fast and thoughtless acceptance of the CDSS decision [6]. From a legal perspective, these effects of practical application of the ESR-iGuide are of great interest, especially the possible question of liability for mistakes that occur based on CDSS usage — the referring physician and/or the radiologist and/or the creators of the CDSS? In the confusing field of medical liability when using CDSS, these systems are already being supported by AI, which will be used even more frequently in the future [7]. At EU level, the legislator has announced that they will create legal certainty for AI applications with the AI Act and AI Liability Directive [8, 9]. It remains to be seen how this turns out in practice. The expected regulated principles within AI context can possibly transfer to CDSS. The quality of AI systems largely depends on how extensive and varied the provided sets of training data are. ESR-iGuide as an expert knowledge is generated randomly over decades, single cases count [10]. For legal handling of CDSS, i.e., for scaling a system and the application at different locations, the fulfilment of all legal requirements at EU level as well as the relevant European member state is essential. In Germany are i.a. medical device regulation, data protection and data security essential.

In any case, referring physicians and radiologists should be aware that generally guidelines are recommendations for action and decision-making, which can or even have to be deviated from in justified cases. The applicability of a guideline or individual guideline recommendations must be checked in every individual situation. When using CDSS, it must be still ensured that the physicians using it ultimately retain their own assessment of each examination and, if in doubt, make a responsible personal decision. Therefore, experience and practical training with the application of CDSS, transparency and comprehensibility of CDSS tool methodologies and decisions are important in medical practice for referring physicians and radiologists. If digital guideline-based systems are to be further developed and applied to the clinical decision-making process in Europe in order to keep improving quality, efficiency, security, transparency and outcomes in clinical radiology, they must also comply with the legal requirements of the European Union (EU) and any special features applicable in the respective EU member states.

## Declarations

### Guarantor

The guarantor of this work is Britta Rosenberg.

### Methodology

• Commentary
